# Assembly, maturation and three-dimensional helical structure of the teratogenic rubella virus

**DOI:** 10.1371/journal.ppat.1006377

**Published:** 2017-06-02

**Authors:** Vidya Mangala Prasad, Thomas Klose, Michael G. Rossmann

**Affiliations:** Department of Biological Sciences, 240 S. Martin Jischke Drive, Purdue University, West Lafayette, IN, United States of America; University of California at Los Angeles, UNITED STATES

## Abstract

Viral infections during pregnancy are a significant cause of infant morbidity and mortality. Of these, rubella virus infection is a well-substantiated example that leads to miscarriages or severe fetal defects. However, structural information about the rubella virus has been lacking due to the pleomorphic nature of the virions. Here we report a helical structure of rubella virions using cryo-electron tomography. Sub-tomogram averaging of the surface spikes established the relative positions of the viral glycoproteins, which differed from the earlier icosahedral models of the virus. Tomographic analyses of *in vitro* assembled nucleocapsids and virions provide a template for viral assembly. Comparisons of immature and mature virions show large rearrangements in the glycoproteins that may be essential for forming the infectious virions. These results present the first known example of a helical membrane-enveloped virus, while also providing a structural basis for its assembly and maturation pathway.

## Author summary

Rubella virus (RV) causes serious fetal defects when contracted during pregnancy. Despite its medical importance, due to the irregular shapes and different sizes of the virions, the RV structure has remained unknown. Using cryo-electron tomography, we have determined the RV structure, which shows a unique, helical outer surface. Subsequent local averaging of the RV surface spikes has established the conformations of its immunogenic glycoproteins. *In vitro* assembly studies on the virus capsid protein have provided insights into the interactions necessary for virus assembly. Comparisons between mature and immature RV show large conformational changes in the virion structure that are essential for virus maturation. These results help to gain a structural understanding of RV pathogenicity, which may also be relevant to other teratogenic viruses.

## Introduction

Rubella virus is an airborne human pathogen that causes a contagious disease with measles-like symptoms in children and adults. Rubella infection in pregnant women can lead to fetal death or severe life-long disabilities such as mental retardation, deafness, cataracts and heart defects in the new-born infants, collectively referred to as congenital rubella syndrome [1]. Despite the availability of an effective vaccine for rubella since the 1960s, the virus is still a global health concern with over 100,000 babies born with congenital rubella syndrome every year [2].

Rubella virus is an enveloped, positive-stranded RNA virus with virions ranging from 500 Å to 900 Å in diameter [3]. The virions form a variety of shapes that range from nearly spherical to elongated tube-like structures [3, 4]. The structural components of rubella virus are comprised of three proteins, glycoproteins E1 (58kDa), E2 (42–47kDa), and the capsid protein (31kDa) [5, 6]. The glycoproteins are type I transmembrane proteins [5, 7] that form heterodimers on the virion surface [8]. E1 is responsible for recognition and attachment to cellular receptors [9]. It is also involved in membrane fusion in the presence of low pH and calcium ions [10–12]. E2 is required for efficient folding and transportation of E1 through cellular compartments [7, 8]. Of the two glycoproteins, only the structure of the E1 ectodomain in its trimeric, post-fusion conformation is known [13]. The rubella E1 ectodomain has an elongated structure, similar to the post-fusion conformations of the alphavirus E1 and flavivirus E glycoproteins [13]. The E1 ectodomain has three domains DI, DII and DIII. DII contains two conserved hydrophobic fusion loops at one end of the rubella E1 structure, whereas DI and DIII form the other end [13]. The third structural protein of rubella virus is the capsid protein, which interacts via its amino-terminal domain with the viral RNA genome to form the inner nucleocapsid [6, 14]. The capsid protein exists as a homodimer [8] and is attached to the viral membrane through the E2 signal peptide [15]. The atomic structure of the carboxy-terminal domain of the capsid protein is known [16] and presumably forms the structural framework of the viral nucleocapsid.

The rubella virus structural proteins assemble on and bud from Golgi membranes [1, 4, 17] in host cells. The newly budded virions form uniformly dense particles within the Golgi and are referred to as ‘immature’ virions [18]. These immature virions undergo structural reorganization during their passage through the Golgi complex to form mature virions that have a double shell-like architecture. The mature virions are then secreted into the extracellular environment [3, 16, 18].

Based on their similar genome organizations, rubella virus and alphaviruses are classified as the only members of the Togavirus family [1, 19, 20]. However, these two groups of viruses have little sequence similarity [21] and differ in their assembly as well as maturation strategies [1, 18]. The alphaviruses include well-known viruses such as Chikungunya virus, Sindbis virus and Semliki Forest virus. They have been studied extensively and their structure determinations have been facilitated by the icosahedral nature of the virions [22–24]. Structural studies have helped in characterizing alphaviruses when complexed with neutralizing antibodies [25–30] and antiviral compounds [31–33]. However, structural information on rubella virus has been minimal owing to the pleomorphic nature of the virus.

In this report, we have used a combination of cryo-electron tomography, sub-tomogram averaging and *in vitro* assembly studies to elucidate the three-dimensional structure and assembly of rubella virions. We have further taken advantage of the direct detector technology to achieve close to nanometer resolution for the sub-tomogram average of the asymmetric and relatively small rubella virus glycoprotein spikes (~100kDa molecular weight). Additionally, we have isolated immature rubella virions that give insights into the different maturation states of rubella virus. Collectively, these analyses suggest a distinctive assembly and maturation pathway for rubella virus that might be critical for the teratogenic pathogenicity of this medically significant virus.

## Results

### Morphology of rubella virions

**Fig 1:**
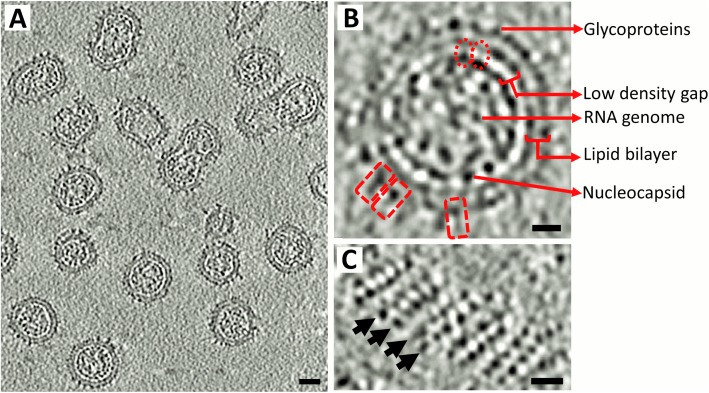
Rubella virion morphology. (A) Section from a rubella virus tomogram showing the different morphologies of rubella virions. Scale bar corresponds to a length of 200 Å. (B) Cross-section of a rubella virion. The dashed rectangles in red show individual rubella glycoprotein spikes. The dashed ovals in red indicate thin densities that connect the inner nucleocapsid shell to the outer glycoprotein plus membrane shell. (C) Surface of a rubella virion showing the glycoprotein rows. Black arrows mark the direction of the rows. Scale bar in panels *B* and *C* correspond to a length of 100 Å. Black is high density in all panels.

Samples of mature rubella virus were prepared for cryo-electron tomography as described previously [3]. About 35 tomograms were collected using the Leginon software [34, 35] and reconstructed using the IMOD software package [36] (Fig 1A, Materials and methods). Distributions of size, shape and morphology of the observed rubella virions (Fig 1A) were similar to previous reports [3, 16]. The virions have an outer shell, about 90–130 Å thick, that includes the glycoproteins and virus membrane. The inner virion shell consists of the capsid protein and the viral genome. The membrane and nucleocapsid are separated on average by about 70 Å [3]. Thin strips of density run across this gap, providing a continuity between the inner nucleocapsid shell and the outer glycoprotein shell [16] (Fig 1B).

### Helical organization of surface glycoproteins

**Fig 2:**
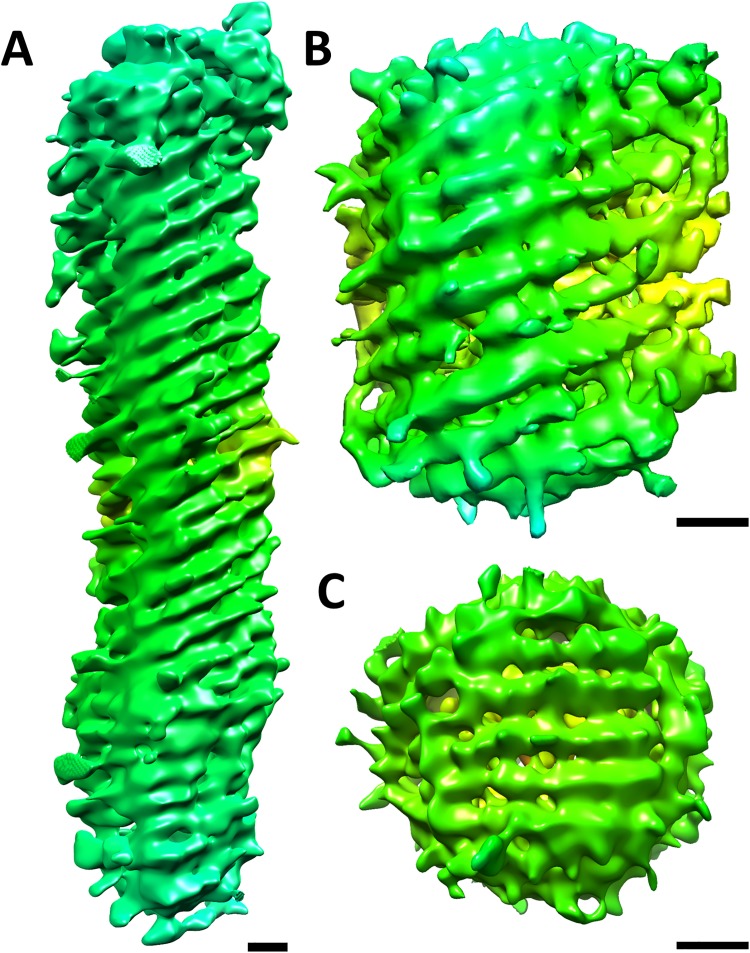
Helical organization of the rubella virus glycoproteins. Representation of three different rubella virions (A, B and C) showing the organization of their surface glycoprotein rows. The virions have been extracted and rendered using UCSF Chimera [61] without any averaging procedures (Materials and methods). The extracted virions have been low pass filtered to 75 Å and hence, the surface glycoprotein rows appear as elevated ridges on the outer membrane surface. Scale bar is 100 Å in length. The surface contour is chosen at 0.81 standard deviations above average. The pitch of the helix in Fig 2A–2C is 533 Å, 390 Å and 0 Å, respectively. Further analysis of the glycoprotein rows using sub-tomogram averaging is shown in S1 Fig.

Variable arrays of surface glycoprotein “spikes” project outwards from the rubella virus membrane [3, 16] (Fig 1B). However, the resolution limits of earlier tomographic studies did not allow characterization of the spatial arrangement of the individual glycoproteins, which can now be delineated with the current tomographic data in this study. The glycoproteins form rows on the virion surface with a separation of 65 Å to 90 Å between rows (Fig 1C). The average separation between glycoprotein spikes is 50 to 55 Å along each row (Fig 1C). The defining characteristic of the rubella virus surface is the tendency to form sets of four to six parallel rows of glycoproteins. In elongated virions, the glycoprotein rows wrap around the virion in a six-start helical pattern that terminate near the ends of the virion (Fig 2A and S1 Movie). Irregular shaped virions that are large and partially tubular also have short regions of helically wrapped glycoprotein rows (Fig 2B and S2 Movie). Small and pseudo-spherical virions appear to be composed of two or three different sets of glycoprotein rows that merge to form a closed shell (Fig 2C and S3 Movie). The helical pitch values are different between individual rubella virions (Fig 2). This irregular helical nature of the glycoproteins on the surface of differently shaped rubella virions was not previously known.

### Structure of the mature glycoprotein spike

**Fig 3:**
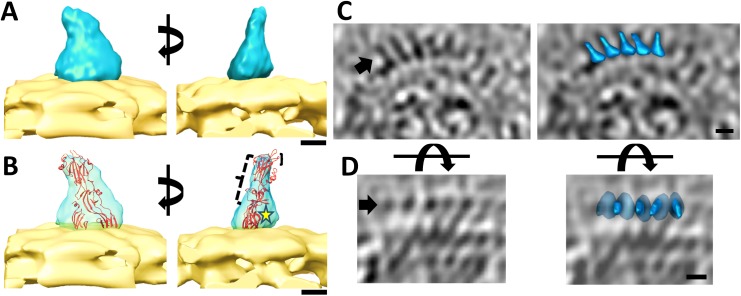
Structure of rubella virus glycoprotein spikes. (A) Sub-tomogram averaged structure of the rubella glycoprotein spike (light blue) is shown placed on a membrane surface (yellow). The membrane surface has been modelled by extracting a lipid bilayer portion from a co-purified membrane vesicle in the un-averaged virus tomograms. The left and right panels are rotated 90° with respect to each other. (B) The same figure as in panel *A*, showing the rubella E1 ectodomain’s atomic structure fitted into the averaged density. The yellow star indicates the location of the rubella E2 ectodomain. The parenthesis in black indicate immunogenic surface regions on E1. Scale bars in panels *A* and *B* correspond to a length of 25 Å. Intermediates from the sub-tomogram averaging procedures are shown in S2 Fig. The Fourier Shell Correlation (FSC) curve calculated to estimate the resolution of the averaged glycoprotein spike map is shown in S3 Fig. See also S1 Table. (C) Cross-section of a rubella virion showing a representative glycoprotein row. Left panel shows the original tomogram section. The right panel shows the same section after placing the averaged glycoprotein spike (blue) (8X binned) into the tomogram. (D) Cross-sections showing a top view of the same glycoprotein row as in panel C. Black arrow indicates the glycoprotein row being considered. In panels C and D, scale bar is 50 Å long and black represents high density.

The rubella glycoproteins (E1 and E2) are present as heterodimeric complexes or spikes on the surface of the virus [3, 8]. However, no direct data was previously available on the relative positions of the rubella surface glycoproteins. To address this lack of information a total of 240 glycoprotein rows containing similarly spaced adjacent spikes from different virions were selected and averaged using the PEET sub-tomogram averaging software [37, 38] (Materials and methods). The resulting averaged map has significant density in only about one half of the volume that represents the base of the spike (S1 Fig). This suggests that the glycoprotein complexes are positioned similarly along a surface row but the external ends of the glycoproteins have different conformations. Consequently, individual glycoprotein spike volumes that appear predominantly straight were picked manually using the IMOD software [36]. The selected sub-volumes were split into two datasets for independent processing and subjected to sub-tomogram averaging procedures (Materials and methods, S2 Fig). The averaged glycoprotein spike structure consists of a broad base below a narrower stalk (Fig 3A) and has an estimated resolution of 11.0 Å (at 0.143 FSC cut-off) and 14.9 Å (at 0.5 FSC cut-off) (S3 Fig). The top (external) part of the averaged glycoprotein spike has weaker density compared to its base, indicating that there is more variability in the region distal from the membrane. The atomic structure of the E1 ectodomain (without it’s stem region (PDB ID: 4ADG) [13]) was fitted into the averaged density using the EMfit program [39] (Fig 3B). A complete three-dimensional search of all possible angles produced three top fits. However, all fits resulted in similar orientations with the long direction of the known E1 structure roughly perpendicular to the viral membrane (S1 Table).

The volume of the averaged spike was 133 Å^3^. From this, the volume occupied by the fitted E1 ectodomain (PDB ID: 4ADG) was subtracted, leaving a residual volume of 52 Å^3^ at the base of the spike. The residual volume can accommodate a protein of roughly 33kDa molecular weight. This is larger than the molecular mass of the E2 ectodomain polypeptide (25kDa) but is smaller than the estimated mass of the glycosylated E2 ectodomain (37kDa to 42kDa). Taking into consideration that the glycosylation on the E2 ectodomain is known to be heterogeneous [5, 40], it is likely that the densities corresponding to the varied carbohydrate moieties on E2 were canceled out during sub-tomogram averaging. Thus, the residual volume at the base of the averaged rubella glycoprotein spike would be sufficient to accommodate the ectodomain of E2 glycoprotein (Fig 3B).

### Local arrangement of the surface glycoproteins

The arrangement of glycoprotein spikes on rubella virions was further examined by placing the sub-tomogram averaged spike, back into each of the original tomographic positions that had been used to obtain the averaged spike structure. The relative orientations of the long axes of the glycoprotein spikes to the viral membrane varied from 30° to 90°. Along glycoprotein rows, adjacent spikes were slightly rotated with respect to each other, along an axis perpendicular to the plane of the membrane. This analysis showed that along unbroken glycoprotein rows in rubella virions, the glycoprotein complexes are similarly oriented with respect to each other, such that E2 would most likely be located between adjacent E1 positions (Fig 3C and 3D).

### Structure of the viral nucleocapsid

**Fig 4:**
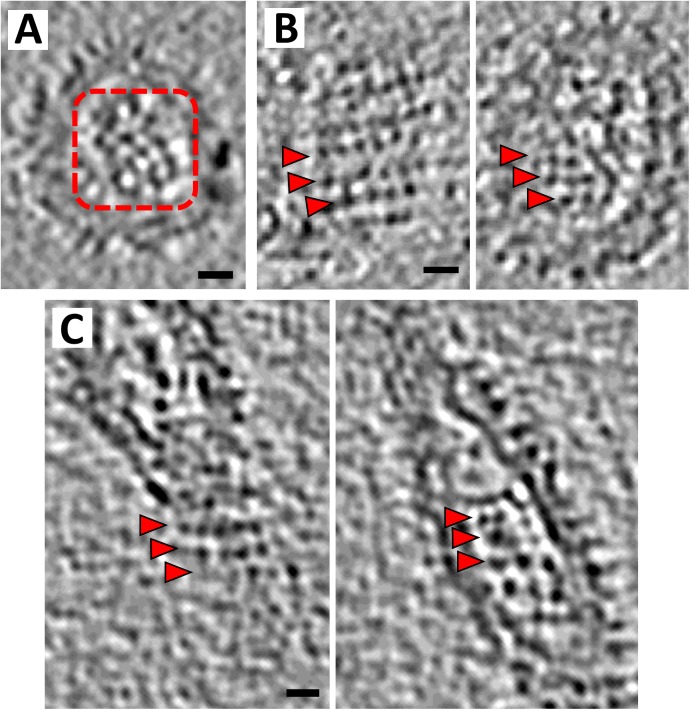
Nucleocapsid organization in rubella virions. (A) Cross-section at the nucleocapsid surface of a rubella virion tomogram, showing a grid-like pattern of the nucleocapsid units (dashed red box). Scale bar is 50 Å long. (B) and (C) Left panel shows a tomogram section at the surface of the rubella virions; the right panel shows a section at the nucleocapsid surface. Red arrows indicate the glycoprotein rows and the corresponding nucleocapsid rows. Scale bar corresponds to a length of 100 Å. Black represents high density. A ball and stick model for the glycoprotein and nucleocapsid organization is given in S4 Fig.

The internal nucleocapsid shell of rubella virus consists of the capsid protein and the viral RNA genome. The nucleocapsid surface follows the contour of the viral membrane [3]. In previous tomographic studies [16], the individual subunits of the nucleocapsid could not be resolved and appeared merely as rows running approximately perpendicular to the glycoprotein rows on the virion surface. In the current tomograms, the nucleocapsid layer appears as globular subunits arranged in a grid-like pattern (Fig 4A). The viral genome is closely associated with the capsid protein leaving a sparsely populated region in the center of the virions. The nucleocapsid layer is more variable than the glycoprotein arrangement on the virion surface, though this could be due to interference from density associated with the viral RNA. The spacing between capsid units in the viral nucleocapsid varies from 40 Å to 70 Å. However, in well-resolved and ordered regions of the virus the nucleocapsid subunits are positioned roughly underneath the surface glycoprotein heterodimers, with each nucleocapsid unit associated with one glycoprotein spike (Fig 4B and 4C, S4 Fig).

### Cryo-electron tomography of *in vitro* assembled nucleocapsid cores

**Fig 5:**
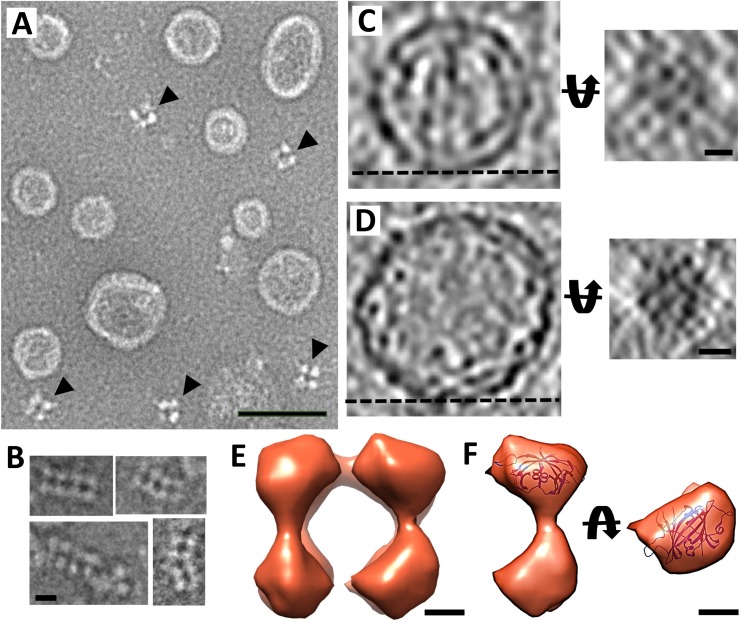
*In vitro* assembled nucleocapsid cores. (A) Negative stain image of purified rubella virus nucleocapsid cores. Black arrows indicate the co-purified capsid tetrameric units. Scale bar is 500 Å long. Agarose gel analysis of the effect of nuclease on the nucleocapsid cores is shown in S5 Fig. (B) Negative stain images of linear assemblies of capsid tetramers. Scale bar is 100 Å long. In panels *A* and *B*, white is high density. (C) and (D) Left panels show tomogram cross-sections from nucleocapsid core particles. Right panels show the end-on view of the nucleocapsid core surface that are indicated in the left panels by a dashed black line. Scale bars in panel C and D are 50 Å and 100 Å long, respectively. Black represents high density. (E) Side view of the sub-tomogram averaged density of the recombinantly produced nucleocapsid tetramers. (F) A single capsid unit density showing the fitted C-terminal domain of the capsid protein. Left panel shows the side view whereas the right panel shows the top view. Scale bar is 25 Å long. A more detailed fitting result for panel F is given in S6 Fig. See also S1 Table.

To differentiate between the viral genome and the capsid protein in the viral nucleocapsid, recombinant rubella virus capsid protein molecules were produced *in vitro* to form nucleocapsid cores (Materials and methods). These *in vitro* assembled rubella virus nucleocapsid cores have a smooth exterior and are hollow. They display a variety of shapes and sizes with diameters ranging from 400 Å to 900 Å (Fig 5A), illustrating that the capsid protein by itself has an inherent tendency to form pleomorphic particles. An agarose gel assay using the purified, recombinant nucleocapsid cores demonstrated that these particles contain nucleic acids of different sizes (S5 Fig). Treatment with benzonase nuclease during cell lysis greatly reduced the yield of the nucleocapsid cores. However, benzonase treatment of purified nucleocapsid cores does not affect the integrity of the cores, but only removes its nucleic acid content (S5 Fig). Thus, benzonase treated nucleocapsid cores were used to remove any interference from nucleic acids for the tomographic studies. Purification of the recombinantly produced capsid protein also resulted in the isolation of a series of intermediate assembled complexes. The smallest identifiable complex consisted of units that appeared to have 4-fold symmetry (Fig 5A). Other larger complexes consisted of linear rows of these tetramers (Fig 5B).

Cryo-electron tomograms of the *in vitro* assembled nucleocapsid cores were collected and processed in a similar manner to the infectious virus samples (Materials and methods). Tomogram sections of the nucleocapsid cores show a tetramer-like pattern as seen in the assembly intermediates (Fig 5C and *5*D). The tomograms also show that these tetrameric arrays have occasional discontinuities that might be necessary to form three-dimensional, closed nucleocapsid core particles (Fig 5D). The arrangement of the capsid subunits in the nucleocapsid cores agrees with the pattern observed in the viral nucleocapsids (Fig 4B and 4C). This implies that the nucleocapsid in rubella virions is composed of a pseudo-tetrameric arrangement of capsid proteins in contact with the viral genome. These observations also confirm that the bulk of the capsid protein lies in the inner shell of the virion, clarifying previous estimates of the capsid protein location [3, 16].

### Sub-tomogram averaging of nucleocapsid units

The cryo-electron density from about 20 isolated tetrameric units seen in the tomograms of the *in vitro* assembled nucleocapsid cores were re-oriented to a common orientation and averaged (Materials and methods). This showed that each monomer of the tetrameric unit has a dumbbell-shaped structure (Fig 5E).

The rubella capsid protein exists as a functional dimer. The C-terminal domain of the capsid protein structure consists of approximately 150 amino acids with 27 amino acids at the C-terminus being disordered in the crystal structure (PDB: 4HBE) [16]. The N-terminal domain consists of approximately 127 amino acids whose atomic structure is unknown. The structure of the C-terminal domain of the capsid protein dimer was fitted into one lobe of the dumbbell shaped averaged density of the *in vitro* assembled nucleocapsid tetramers using the EMfit program [39] (Fig 5F, S1 Table). The two top fitting orientations for the capsid protein in the averaged density are 180° apart, relative to each other, along an axis perpendicular to the two-fold axis of the capsid dimer (S6 Fig, S1 Table). These best fitting conformations of the capsid protein are similar to the orientations of the capsid protein expected to be facing the viral membrane [16]. As the N- and C-terminal domains of rubella capsid protein are similar in size, the remaining density of the averaged dumbbell shaped unit must be the location of the N-terminal domain of the capsid protein.

Using these fitting results, it can be deduced that the disordered residues at the C-terminal end of the capsid protein, not seen in its crystal structure [16], likely correspond to the thin strips of density visible in the cross-section of rubella virion tomograms (Fig 1B), linking the inner nucleocapsid shell to the membrane anchored E2 signal peptide in the viral membrane. The dumbbell shape of the capsid protein also accounts for the double-layer appearance seen in the *in vitro* nucleocapsids (Fig 5C and 5D). In the virions, there are long pieces of density, which correspond to the viral RNA, that are closely associated to the N-terminal region of the capsid proteins. Thus, the double layer characteristic of the nucleocapsid is not as obvious in the virions as it is in the *in vitro* nucleocapsid cores.

### Structural implications from rubella virion maturation

**Fig 6:**
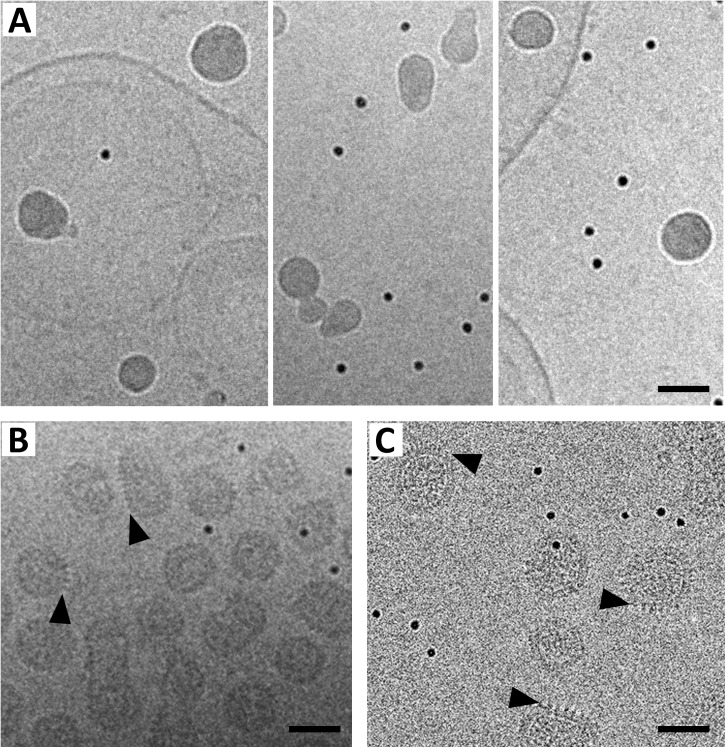
Cryo-electron microscopy image of purified immature rubella virions. (A) The approximately 500 Å diameter, uniformly dense and smooth particles in the images represent the immature virions captured on a 1k×1k CCD camera with a dose of 20 e^-^/Å^2^. (B and C) Zero degree tilt images of mature rubella virions collected using a 2k×2k CCD camera (dose: 2e^-^/Å^2^) and with a Gatan K2 Summit direct electron detector (dose: 0.8e^-^/Å^2^) respectively. The spiky nature of the mature rubella virions (indicated by black arrow heads) is seen even under the very low dose images in panels B and C. All panels also contain 100 Å BSA-gold particles (small and very dense particles). Scale bar is 500 Å long. Black represents high density.

Vero cells infected with rubella virus were lysed at 22 hours’ post-infection to release intracellular virion particles. Further virus purification was carried out using the cell lysates (Materials and methods). Cryo-electron microscopy of the purified, immature virus particles shows that the immature virions are uniformly dense and variable in size (Fig 6A) corroborating earlier descriptions of immature rubella virions [18]. The immature virions have a smooth exterior with no prominent features (Fig 6A), which implies that during the early stages of virion budding and transport inside host cells, rubella E1 lies close to the virion surface instead of protruding out from the surface as in extracellular mature virions (Fig 6B and 6C). The uniform dense nature of the immature virions also suggests that in the initial immature state, the glycoproteins and membrane layer are more closely interacting with the nucleocapsid layer than they are in the mature virions. Hence, the glycoprotein and nucleocapsid layers assemble probably into a more compact arrangement in the initial immature form, with the glycoproteins in register with the capsid proteins. Loss of order could be a product of structural reorganization that occurs during virion maturation.

## Discussion

The rubella virus structure had been expected to have T = 3, icosahedral symmetry [1, 8, 41]. Instead, the structure of rubella virus, as shown here, has an irregular helical organization of its surface glycoproteins and a pseudo-tetrameric inner nucleocapsid arrangement. The glycoprotein arrangement in rubella virions is unique, as other known membrane enveloped viruses exhibit helical structures only in their inner nucleoprotein complex or in their matrix protein layer, such as in paramyxoviruses [42], marburgviruses [43], and influenza-A viruses [44]. Thus, rubella virus is the only known example of a helical surface structure associated with a membrane enveloped virus.

The relative positions of the rubella glycoproteins, with an extended E1 conformation and with E2 at the base of the spike complex, is different from the glycoprotein heterodimer conformation observed in alphaviruses. However, this structural placement of rubella glycoproteins is consistent with protease studies [10, 45] and immunological reactivity studies on rubella virus [46, 47], which indicate that the E1 molecule is more exposed and accessible than E2 on the virion’s surface. E1 is the primary target for neutralizing antibodies against rubella virus. The common antibody binding region on E1 (between residues 202 to 285) [48–52] is exposed to the surrounding environment in the spike density, given the orientation of E1 as determined by the fitting results. Though the E1 crystal structure used in this study is similar to the post-fusion E1 and E glycoprotein structures of alpha- and flaviviruses, the rubella E1 structure was determined under neutral pH and in the absence of detergents [13], unlike in the case of the post-fusion structures of alphavirus E1 [53] and flavivirus E glycoproteins [54]. Moreover, the translation of domain III between the pre- and post-fusion conformations in alpha- and flaviviruses, is possibly a consequence of the large conformational change of the glycoproteins from being tangential to being almost perpendicular to the viral membrane. Under these considerations, the proposed movement of domain III in alpha- and flaviviruses might not be directly applicable to rubella virus. Even with a possible translational movement of domain III, the position of the volume assigned to rubella E2 would not change significantly. Furthermore, expression of rubella E1 and E2 ectodomains together results in secretion of the E1 ectodomain alone [13], indicating that the E2 ectodomain has a higher affinity to membranes than E1. Thus, these observations are consistent with the placement of E2 close to the base of the rubella glycoprotein spike, rendering it relatively inaccessible compared to E1. The molecular weight of rubella virus E2 is only about one half of E2 in alphaviruses. There is also no significant sequence similarity between the E2 proteins of rubella and alphaviruses. Hence, the structure of E2 in these viruses is probably quite different.

In alphaviruses, the glycoprotein E1-E2 heterodimers lie close to the membrane surface in their pre-fusion state with the E1 fusion loop physically masked by the neighboring E2 molecule. In the rubella glycoprotein complex, the E1 protein extends out from the virion surface and does not appear to require E2 for shielding its two fusion loops. The rubella virus E1 crystal structure [13], which was determined in the absence of detergent and at neutral pH, also presents an extended E1 conformation with fusion loops exposed but not positioned for membrane insertion. The observed variability in the glycoprotein spikes suggest that the tips of the E1 structures are flexible, which would help E1 to avoid non-productive membrane interactions at neutral pH. This might also help explain the requirement for calcium ions to stabilize the rubella E1 fusion loop conformations for membrane insertion at low pH [12, 13].

Difference in the glycoprotein spike orientations between the immature and mature virion forms could also be to protect the fusion loops on the E1 glycoprotein structure from undergoing unproductive membrane interactions in the low pH environment of the cellular Golgi network. Subsequent virion maturation in the Golgi complex would then be necessary for reorganization of the viral envelope to yield fusion competent, extracellular, ‘spiky’ mature virus particles. A similar strategy of surface glycoprotein reorganization during virion maturation occurs also in bunyaviruses [55] and flaviviruses [56].

In addition to the glycoprotein arrangement, the association of protein tetramers to form enclosed shells as seen in the rubella virus nucleocapsids is also unusual and not observed in other known virus structures. Rubella capsid protein molecules expressed in bacterial cells, spontaneously form nucleocapsid cores in the presence of cellular nucleic acids. This indicates that the rubella capsid protein does not need the rubella genome for nucleocapsid formation, similar to previous observations [57], but can associate with random nucleic acids to form nucleocapsids. In the context of virus assembly, this implies that the binding of the capsid protein to the viral RNA is not entirely sequence specific. This suggests that the virus machinery uses other methods, such as an abundance of viral RNA relative to cellular RNA at the virus budding site [58], for efficient packaging of the viral genome into budding virions.

Thus, rubella virus appears to share certain common characteristics, not only with alphaviruses but also with other arbovirus genera, such as flaviviruses and bunyaviruses. However, in addition to these features that suggest similarities to arboviruses, rubella virus has also evolved some unique traits such as an exposed E1 fusion loop conformation. Together, these observations point to a more complex structural evolution in rubella virus than previously assumed.

## Materials and methods

### Preparation of virus samples

Mature rubella virus (M33 strain) was cultured in Vero cells (ATCC CL-81) and purified according to previously published protocols [3]. For purification of intracellular immature rubella virions, a modified protocol similar to purification of intracellular retroviruses [59] was followed. Briefly, rubella virus (M33 strain) infected Vero cells were lysed 22 hours’ post-infection by suspending the cells in a hypotonic buffer containing 20mM Tris pH 8.0, 10mM NaCl plus 15mM MgCl_2_ and then homogenizing the suspension in a Dounce homogenizer. The cell lysates were centrifuged at 3000×g for 5min to pellet cell debris. The supernatant was further centrifuged at 20,000×g for 20 min. The supernatant was again collected and incubated with 5mM EDTA for 10min followed by addition of 10μg/ml of RNase A. After 15 minutes, the supernatant buffer concentration was adjusted to contain 120mM NaCl. The supernatant containing the immature virions was further purified similar to the mature virus purification protocol [3]. A discrete band in the expected virus density range was observed after density gradient ultracentrifugation, which was then extracted for cryo-EM analysis. Mock-infected Vero cells were lysed and treated in the exact same manner as the rubella virus infected Vero cells during immature virion purification. No bands were seen in the density gradient after the final gradient ultracentrifugation step in the mock-infected control.

### *In vitro* assembly of rubella nucleocapsids

The rubella virus capsid gene has two methionines in the first 10 nucleotides of its sequence (at positions 1 and 9). To improve bacterial expression, the nucleotide sequence corresponding to amino acids 1 to 8 was removed. Rubella virus (M33 strain) capsid protein (RVC) gene sequence corresponding to amino acids 9–277 was cloned into a pTXB1 plasmid (New England Biolabs). The RVC-9-277-pTXB1 construct was transformed into *E*. *coli* BL21 (DE3) cells (Novagen). Cells were grown at 37°C and induced with isopropyl thiogalactoside (IPTG) when the O.D reached 0.8. The induced cultures were grown at 25°C for 5 hours and pelleted. Cell pellets were re-suspended in buffer containing 50mM Tris (pH 7.2) and 500mM NaCl. The cells were lysed using sonication and the target protein purified using a chitin column (New England Biolabs). The purified RVC-9-277 protein was applied to a Sepahcryl S-400 column (GE Healthcare) to fractionate the sample according to different apparent molecular weights. Fractions corresponding to assembled nucleocapsids and smaller capsid protein complexes were pooled and concentrated separately. The nucleocapsid core samples were treated with 0.001% benzonase and the buffer was exchanged before sample preparation for cryo-electron tomography.

### Cryo-electron microscopy and sub-tomogram averaging

For cryo-electron tomography, the purified samples were mixed with 4X concentrated 10nm BSA gold solution (Aurion, Wageningen, Netherlands) in a 15:1 volume ratio. 3μl of this mixture was applied to Quantifoil R1.2/1.3 grids (Electron Microscopy Sciences), blotted and plunge frozen in liquid ethane.

Tilt-series data was collected using a Titan Krios (operated at 300kV) and a Gatan K2 Summit detector. The Leginon software [34, 35] was used to collect data at appropriate grid positions. A magnification of 11000X was used with a pixel size of 1.32Å. Tilt-series were collected from -60° to +60° in 1.5° steps with a defocus range of 4–5μm for the virus samples and 5–6μm for the *in vitro* assembled nucleocapsid core samples. The total dose applied to each sample was between 80–90 e^-^/Å^2^ [34, 35].

The tilt-series were aligned using gold fiducial markers and reconstructed using the IMOD software [36]. Ctfplotter program from the IMOD suite [36] was used to estimate the defocus of each individual tilt-image in the tilt-series. CTF correction and phase-flipping of the aligned tilt-series was then carried out as part of the IMOD tomographic reconstruction procedure. Though accurate resolution estimation is challenging for tomographic reconstructions, the resolution of the virus tomograms was estimated to be better than 50 Å because it was possible to see separation between the two layers of the viral membrane in directions perpendicular to the electron beam.

For negative staining, 1% solution of ammonium molybdate was added to the sample on a grid, blotted, washed twice with distilled water and then air dried after blotting off the excess liquid. The grids were analyzed using a CM200 (FEI) microscope with a 1k×1k CCD camera.

### Preparation of individual virion movies

Sub-volumes of virions were extracted from the cryo-electron tomograms using IMOD [36]. The representation of the virion density was inverted using the EMAN2 suite [60]. Using the UCSF Chimera software [61], the virion density maps were low pass filtered using a gaussian filter to 75 Å resolution, followed by removal of dis-continuous densities using the ‘hide dust’ feature. The virions were then rotated around different orthogonal axes for making a movie using Chimera [61]. Snapshots from these movies in color were used to make Fig 2A–2C.

### Sub-tomogram averaging procedures

For sub-tomogram averaging of individual glycoprotein spikes that were elongated and predominantly straight, 7290 sub-volume positions were picked on the surface of virions in all directions from 8X binned tomogram data using IMOD [36]. Each glycoprotein spike was identified using two points. The first point was placed close to the spike end which is distal to the membrane and the second point to the spike end closer to the membrane. Only spikes that looked predominantly straight were picked from the tomograms, such that a line connecting the two points on the spike, passed essentially through the center of the spike stalks. The program ‘stalkInit’ in the PEET suite of programs was then used to convert these points into corresponding ‘motive lists’ with position co-ordinates for each spike as input into the PEET suite. Rotation angles were calculated for each spike as the angles between the lines indicating the spikes and the tomogram ‘y’ axis. Subsequent sub-tomogram extraction and averaging procedures were carried out with the PEET software [37, 38] using CTF corrected, 4X binned data.

As the spikes on the virion surface are densely packed and touch each other at the base, a tight cylindrical mask with a radius of 6 pixels (31.6 Å) and a soft Gaussian fall-off of 6 pixels was applied to the particles such that only one spike was visible in the extracted sub-volumes. Before starting the alignment and averaging procedure, all the particles were rotated to align the long axis of the spikes (given by the two identification points) in one common direction. Individual particles were checked to make sure that all the spikes were oriented in the same direction with no inversion of the spikes’ orientations. The search parameters allowed for a complete 360° search about the long axis of the spikes, but restricted the search in the other two directions to ±30° with 0° being along the long direction of the spikes. The initial alignment iterations were coarse searches followed by finer interval searches in later iterations. Missing wedge compensation feature within the PEET software [37, 38] was applied during alignment and averaging of the sub-tomogram volumes.

Initially, five spikes that looked properly formed from different tomograms were selected and independently used to align 256 sub-volumes to calculate initial models. The model which best resembled the individual spike conformations was selected for further steps. The remaining sub-volumes were split into two halves and processed independently. The initial model was used as the reference model for aligning the individual spikes at the first iteration. For subsequent iterations, the reference model was updated by averaging spikes with the highest correlation coefficients to the reference model in the previous iteration, representing 2/3^rd^ of all the spikes. Sub-volumes were aligned to the updated reference model at each iteration. After the final iteration, the determined relative position and angles for each sub-volume was used to average all the sub-tomograms together to give an averaged 4X binned map of the glycoprotein spike. The determined relative orientations and positions were then applied to 2X binned data and the final sub-tomogram average map was calculated using 2X binned data with a pixel size of 2.64 Å. The reference models at every alternate iteration along with the final averaged map are shown in S2 Fig.

The final 2X binned averages from the two half-sets were applied with a soft-edged mask that covered only the glycoprotein spike in order to exclude the surrounding, weak membrane densities. A Fourier-Shell Correlation (FSC) curve was subsequently calculated between the two masked averages using the EMAN2 software suite [60]. The sub-tomogram averaged map was subsequently low pass filtered to 8 Å and then sharpened with an ad hoc B-factor of—375 Å^2^ to increase the impact of higher resolution terms in the map. This sharpened map was used for fitting of the E1 crystal structure.

For sub-tomogram averaging of rows of glycoproteins (as opposed to individual spikes), 240 rows containing four spikes each, with approximately similar spacing between the spikes, were picked using IMOD [36] from 8X binned data. The aligning and averaging procedures were performed with the PEET software [37, 38] using 4X binned data. Four different rows were used as independent starting models. The sub-volumes were aligned against the starting models for iterative refinements and averaged. All the independent averaged results had very similar final density.

Sub-tomogram averaging of the recombinantly produced capsid tetramers were also performed in a similar way to the above described routine for glycoprotein rows. Twenty tetramer sub-volumes were picked from 8X binned tomograms of nucleocapsid cores. Alignment and averaging was performed using 4X binned data.

## Supporting information

S1 Table Fit of atomic structures to sub-tomogram averaged density. (DOCX)

S1 Fig Surface representation of the averaged density of rubella virus glycoprotein rows (in blue). The atomic model of the rubella E1 glycoprotein (red) is placed into one of the subunits of the averaged density to show that the averaged volume of the rubella glycoprotein rows only covers about half the volume of the E1 structure. The left and right panels are rotated 90° with respect to each other. Scale bar is 50 Å long.
(TIF)

S2 Fig Glycoprotein spike intermediate volumes from the sub-tomogram averaging procedure. From top left to bottom right: The top left panel shows the initial model derived from 256 initial spikes. The next three panels show the updated reference models derived from 2/3^rd^ of the total sub-volumes for iterations 3, 5 and 7. The bottom middle panel shows the raw final average derived for the 4X binned data. The bottom right panel shows the raw final average for 2X binned data. The black arrows indicate the positions of the top and bottom of the membrane region. Scale bar in black indicates 25 Å.
(TIF)

S3 Fig Resolution of the sub-tomogram averaged glycoprotein spike. Fourier Shell Correlation (FSC) curve (gold-standard) between the masked averages from two independent half-sets of the glycoprotein spikes. The dashed line at 0.143 FSC intersects the curve at 11.0 Å.
(TIF)

S4 Fig Model representing the relationship between glycoproteins on the virion surface and the internal nucleocapsid units. (A and B) The virions represented here are the same as in Fig 4B and 4C, respectively. Left panel shows a tomogram section at the surface of the rubella virions; the right panel shows a section at the nucleocapsid surface. Scale bars correspond to a length of 100 Å. Black represents high density. Red arrows indicate the glycoprotein rows and the corresponding nucleocapsid rows. The glycoprotein units are indicated as blue rings and the corresponding nucleocapsid units are indicated as orange rings. Underneath the tomogram panels, the glycoproteins and corresponding nucleocapsid units indicated in the tomogram sections are represented as ball and stick models. The blue and orange colored spherical balls represent the positions of the glycoprotein base near the viral membrane and the nucleocapsid units respectively. Units forming a row in the tomograms are connected by lines in the models. Top and side views of the models are shown to indicate the one-to-one relationship between the glycoprotein and nucleocapsid units.
(TIF)

S5 Fig Agarose gel analysis of *in vitro* assembled nucleocapsid cores. Left panel is an agarose gel under UV illumination to show the presence of nucleic acids. The right panel shows the same gel after Coomassie blue staining to indicate presence of protein. Lane 1: 1kb DNA ladder, Lanes 2 and 4: purified nucleocapsid cores, Lanes 3 and 5: purified nucleocapsid cores after benzonase treatment. Capsid protein in lane 4 (in the presence of nucleic acid) appears as a diffused band whereas in lane 5 the protein band is discrete. Black brackets indicate the protein regions in the stained gel.
(TIF)

S6 Fig Fitting of the capsid protein’s C-terminal domain into the sub-tomogram averaged capsid density. Left and right panel show the two best fit orientations of the capsid protein structure into one lobe of the capsid unit density.
(TIF)

S1 Movie Surface representation of an unaveraged long rubella virion. (MP4)

S2 Movie Surface representation of an unaveraged, partially tubular rubella virion. (MP4)

S3 Movie Surface representation of an unaveraged, small, pseudo-spherical rubella virion. (MP4)
