# Supplementary material for: Assembly, maturation and three-dimensional helical structure of the teratogenic rubella virus
Source: PLoS Pathog. 2017 Jun 2;13(6):e1006377. doi: 10.1371/journal.ppat.1006377 (PMC5470745; doi:10.1371/journal.ppat.1006377)
Supplement: S1 Table — (DOCX) [file ppat.1006377.s001.docx]

**S1 Table. Fit of atomic structures to sub-tomogram averaged density.**

|  | EMfit (sumf value^a^) |
| --- | --- |
| Fitting of E1 (PDB ID: 4ADG) into averaged glycoprotein spike | 36 |
| Fitting of C-terminal domain of capsid protein (PDB ID: 4HBE) into averaged capsid unit | 66  66^b^ |

^a.^Sumf values indicate the mean density of all atoms when fitting into the cryo-EM density normalized with respect to the highest density within the map.

# ^b^ Each fit of a complete three-dimensional search refined to the two best fits. These fits were 180.0° apart about a common axis.
