# Supplementary figures and images for: Assembly, maturation and three-dimensional helical structure of the teratogenic rubella virus
Source: PLoS Pathog. 2017 Jun 2;13(6):e1006377. doi: 10.1371/journal.ppat.1006377 (PMC5470745; doi:10.1371/journal.ppat.1006377)

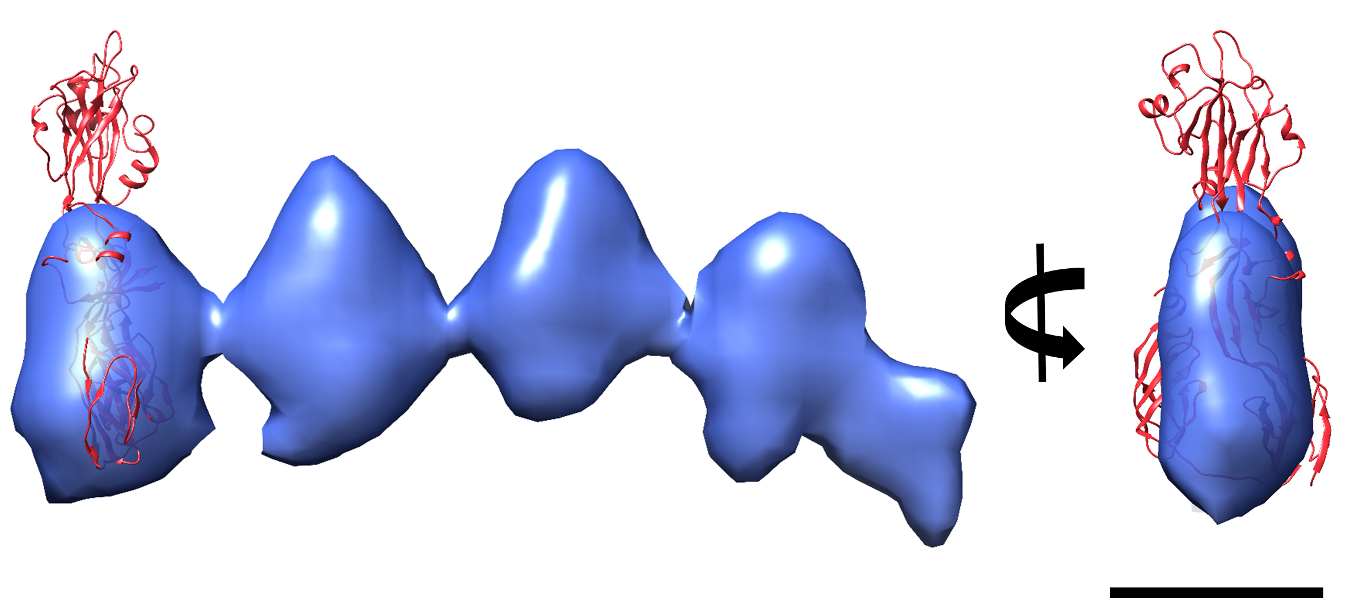

Supplement: S1 Fig — The atomic model of the rubella E1 glycoprotein (red) is placed into one of the subunits of the averaged density to show that the averaged volume of the rubella glycoprotein rows only covers about half the volume of the E1 structure. The left and right panels are rotated 90° with respect to each other. Scale bar is 50 Å long. (TIF) [file ppat.1006377.s002.tif]

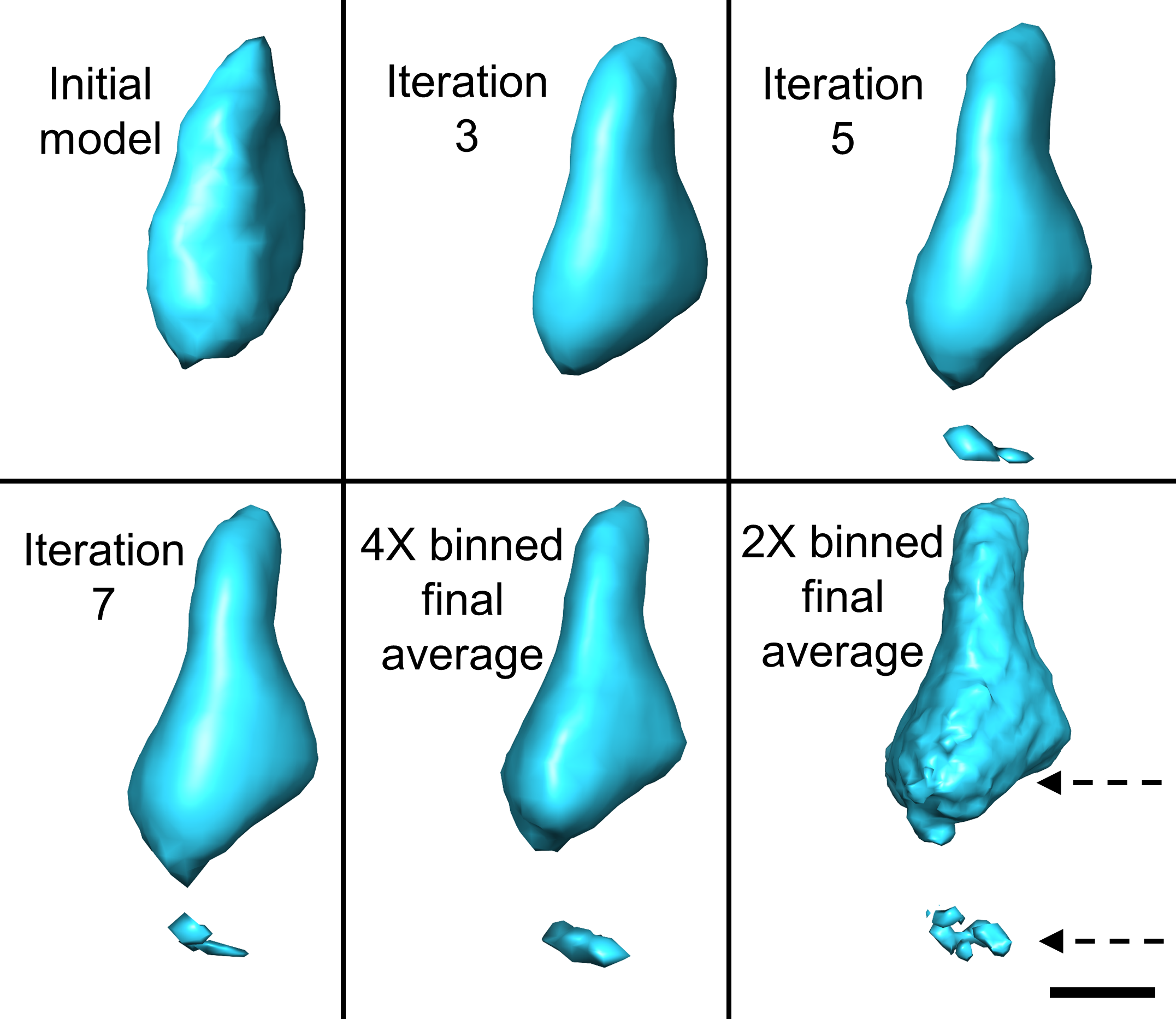

Supplement: S2 Fig — From top left to bottom right: The top left panel shows the initial model derived from 256 initial spikes. The next three panels show the updated reference models derived from 2/3rd of the total sub-volumes for iterations 3, 5 and 7. The bottom middle panel shows the raw final average derived for the 4X binned data. The bottom right panel shows the raw final average for 2X binned data. The black arrows indicate the positions of the top and bottom of the membrane region. Scale bar in black indicates 25 Å. (TIF) [file ppat.1006377.s003.tif]

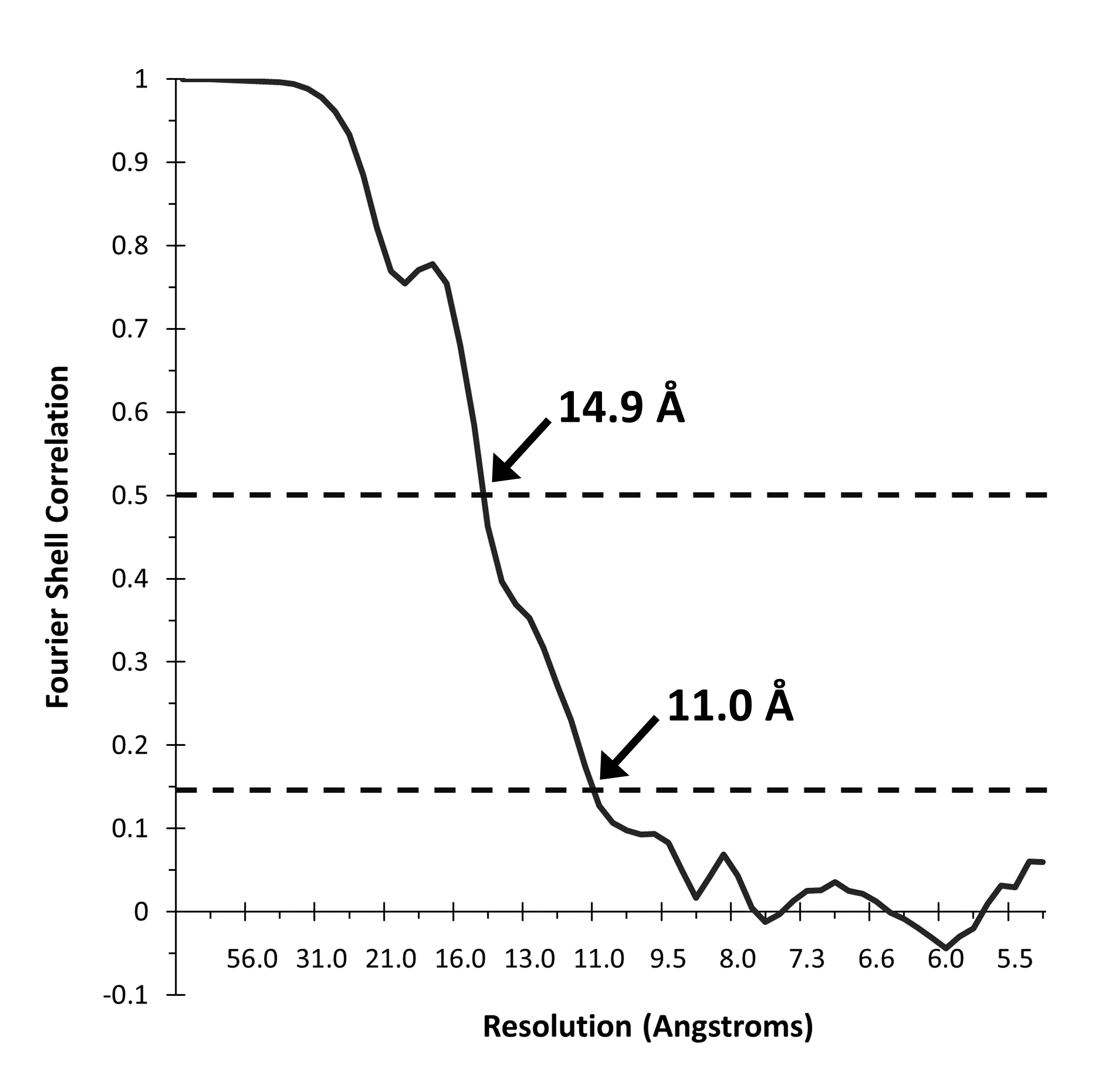

Supplement: S3 Fig — Fourier Shell Correlation (FSC) curve (gold-standard) between the masked averages from two independent half-sets of the glycoprotein spikes. The dashed line at 0.143 FSC intersects the curve at 11.0 Å. (TIF) [file ppat.1006377.s004.tif]

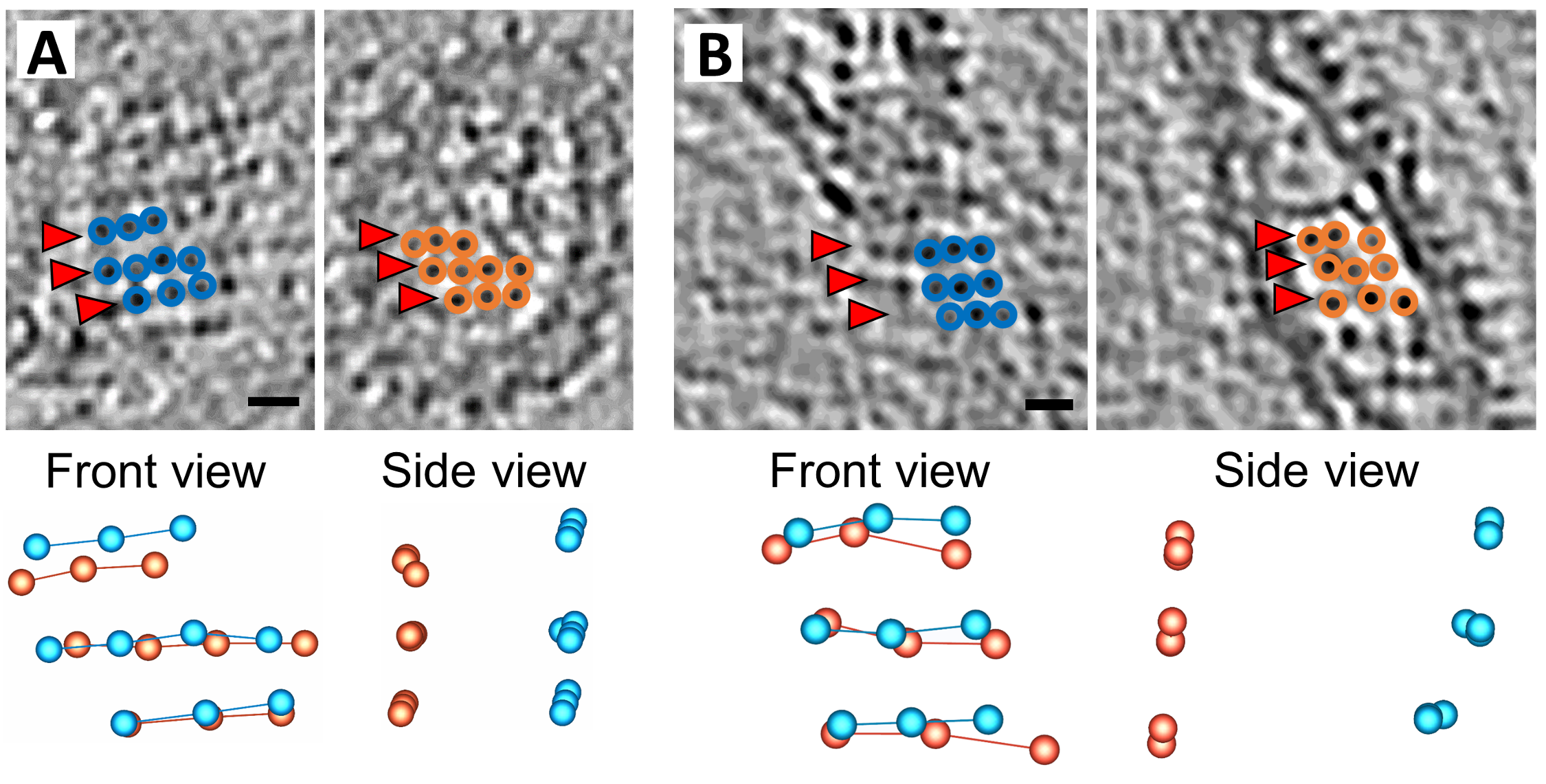

Supplement: S4 Fig — (A and B) The virions represented here are the same as in Fig 4B and 4C, respectively. Left panel shows a tomogram section at the surface of the rubella virions; the right panel shows a section at the nucleocapsid surface. Scale bars correspond to a length of 100 Å. Black represents high density. Red arrows indicate the glycoprotein rows and the corresponding nucleocapsid rows. The glycoprotein units are indicated as blue rings and the corresponding nucleocapsid units are indicated as orange rings. Underneath the tomogram panels, the glycoproteins and corresponding nucleocapsid units indicated in the tomogram sections are represented as ball and stick models. The blue and orange colored spherical balls represent the positions of the glycoprotein base near the viral membrane and the nucleocapsid units respectively. Units forming a row in the tomograms are connected by lines in the models. Top and side views of the models are shown to indicate the one-to-one relationship between the glycoprotein and nucleocapsid units. (TIF) [file ppat.1006377.s005.tif]

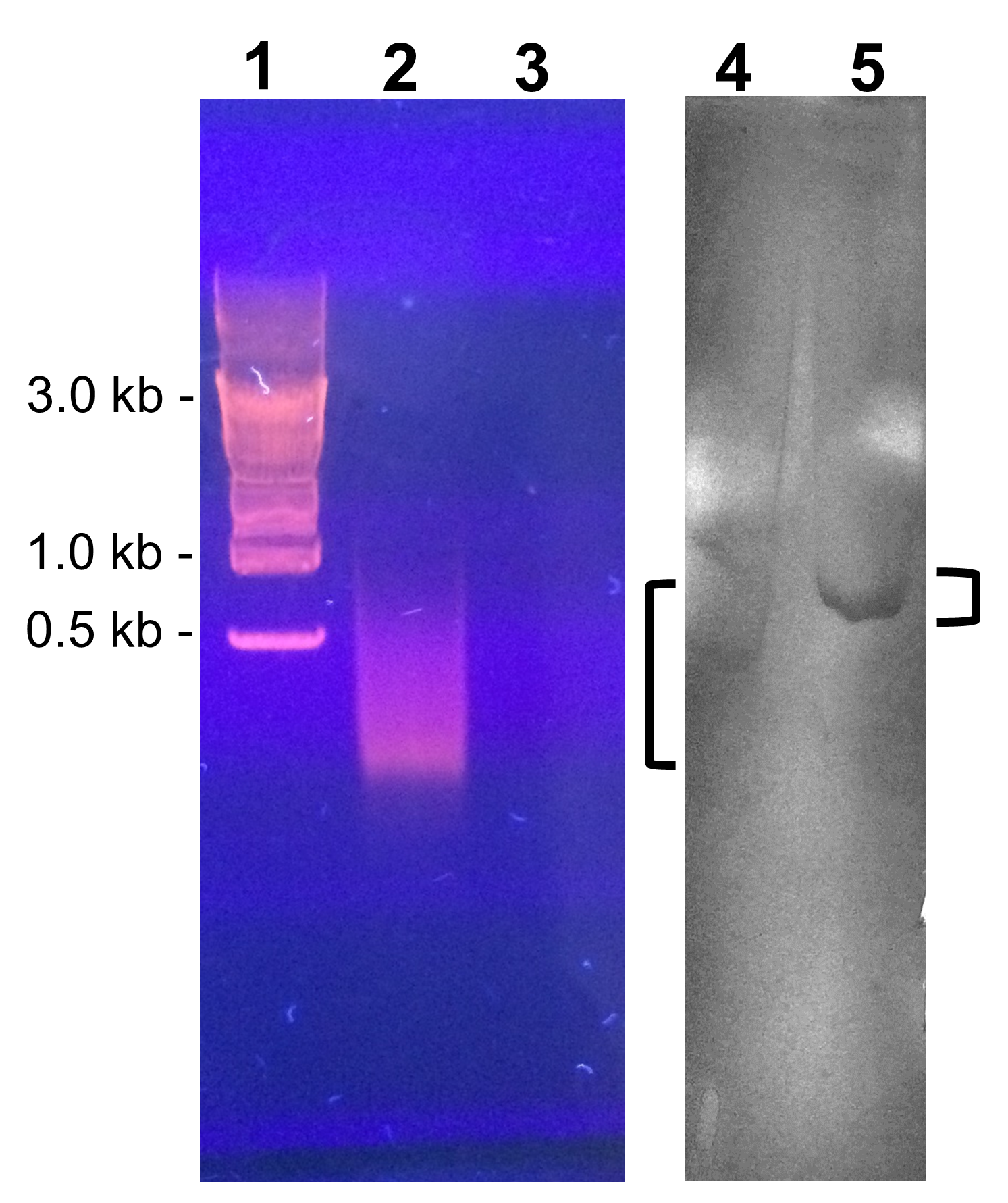

Supplement: S5 Fig — Left panel is an agarose gel under UV illumination to show the presence of nucleic acids. The right panel shows the same gel after Coomassie blue staining to indicate presence of protein. Lane 1: 1kb DNA ladder, Lanes 2 and 4: purified nucleocapsid cores, Lanes 3 and 5: purified nucleocapsid cores after benzonase treatment. Capsid protein in lane 4 (in the presence of nucleic acid) appears as a diffused band whereas in lane 5 the protein band is discrete. Black brackets indicate the protein regions in the stained gel. (TIF) [file ppat.1006377.s006.tif]

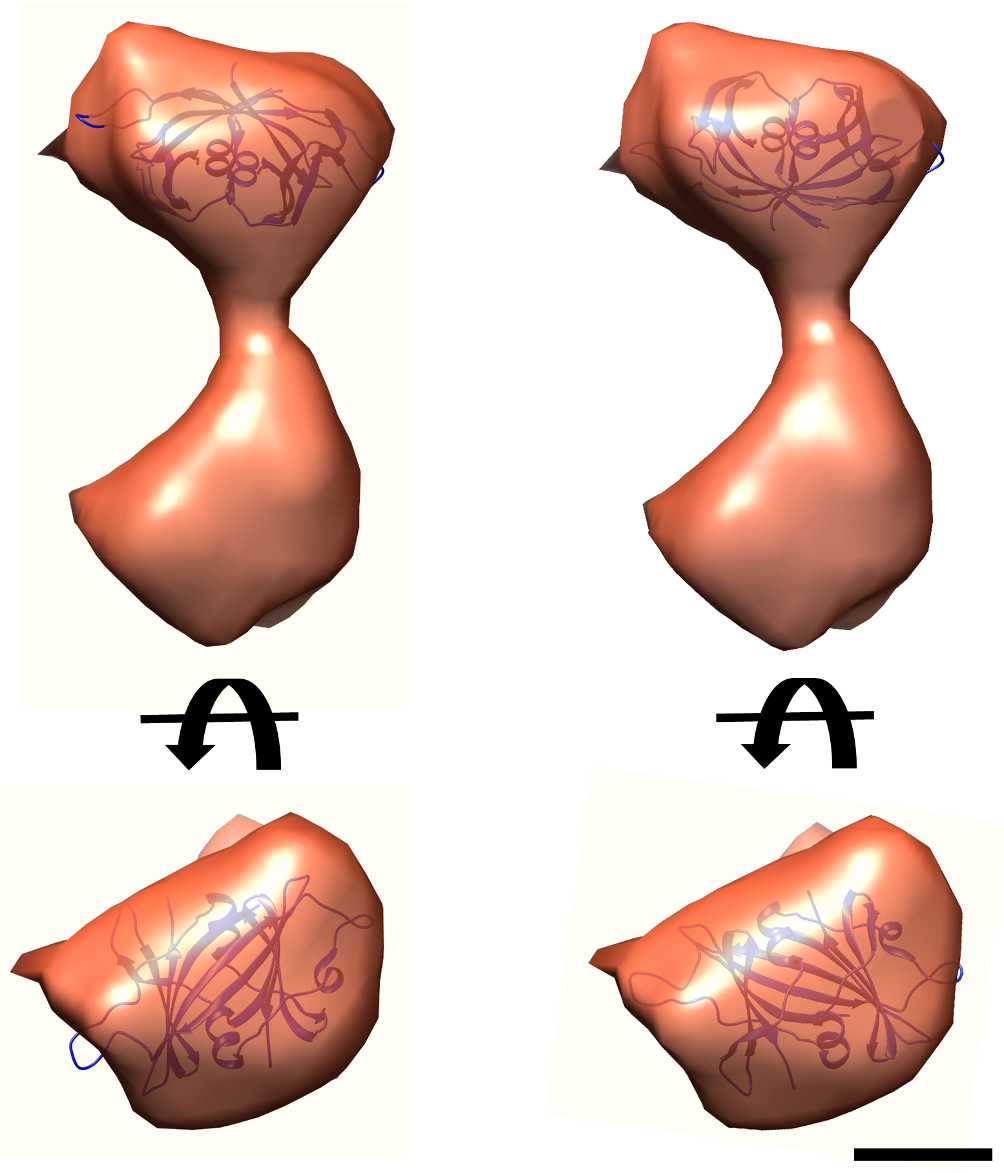

Supplement: S6 Fig — Left and right panel show the two best fit orientations of the capsid protein structure into one lobe of the capsid unit density. (TIF) [file ppat.1006377.s007.tif]
